# Long-Term Persistence of COVID-Induced Hyperglycemia: A Cohort Study

**DOI:** 10.4269/ajtmh.22-0695

**Published:** 2024-02-13

**Authors:** Vrinda Goel, Alpana Raizada, Amitesh Aggarwal, SV Madhu, Rajarshi Kar, Ananya Agrawal, Vikash Mahla, Ashish Goel

**Affiliations:** ^1^ Department of Medicine, University College of Medical Sciences, Delhi, India;; ^2^ Department of Endocrinology, University College of Medical Sciences, Delhi, India;; ^3^ Department of Biochemistry, University College of Medical Sciences, Delhi, India;; ^4^ Hamdard Institute of Medical Sciences and Research, Delhi, India;; ^5^ Department of Medicine, Ambedkar State Institute of Medical Sciences, Sahibzada Ajit Singh Nagar, Punjab, India

## Abstract

Although the short-term mortality of patients with COVID-19 infection and hyperglycemia has been well documented, there is little available data regarding longer-term prognosis. The presence of diabetes has not only influenced disease severity but has also impacted its transmission dynamics. In this study, we followed a historical cohort of patients without previous history of diabetes who presented with moderate to severe COVID-19 and were found to have hyperglycemia (random blood glucose > 140 mg/dL) at the time of admission. We evaluated the need for antidiabetic therapy in these patients at the end of 6 months and the risk factors associated with persistent hyperglycemia determined by monthly values of self-monitored blood glucose. Of the seventy participants who were followed telephonically, 54 (77%) continued to receive antidiabetic therapy or have persistent hyperglycemia (> 140 mg/dL) at the end of 6 months. Persistent hyperglycemia at the end of follow-up, was found to be associated with a higher blood glucose at presentation.

## INTRODUCTION

The seemingly parallel lines for communicable and noncommunicable disease patterns do not appear as distinct as they once did. The increasing overlap calls for a rethink of our strategies to equip our healthcare systems to deal with the emerging interrelations between these disease states. This overlap has recently been underscored by the reports of associations reported between COVID-19 and diabetes. Not only did presence of diabetes influence the severity and outcomes of the disease, but it also influenced transmission dynamics of disease.

Although several theories have been proposed for the association seen between COVID-19 infection and hyperglycemia, the literature remains ambiguous about the bidirectional relationship between these conditions. Presence of diabetes was earlier associated with an increased risk of infection with Middle East respiratory syndrome and SARS CoV-1.^1^ The significance of angiotensin converting enzyme 2–mediated entry of SARS CoV-2 into pancreatic cells and whether this leads to autoimmune destruction of islet cells causing insulin dependence and resistance has been proposed^2^ but remains debatable. It has been postulated that cytokine release during SARS-CoV-2 infection may precipitate the onset of metabolic alterations by affecting glucose homeostasis.^3^ The various hypotheses involved include unmasking of previously existing disease, manifestation of stress, hypoxic lung injury or a direct destruction of pancreatic cells, and also steroid-induced hyperglycemia.^4^

Several similarities have been observed in the pathophysiology of diabetes and infection with COVID-19. It is known that diabetes is associated with immune dysregulation, enhanced inflammation, and impaired beta cell function.^5^^,^^6^ It is a common comorbidity in patients with chronic obstructive lung diseases, idiopathic pulmonary fibrosis, or bronchial asthma aggravating the severity of lung injury in the form of microvascular injury.^7^ It has been observed that patients with COVID-19 have lower lymphocytes and raised neutrophils. Pro-inflammatory markers such as high CRP (C-reactive protein), procalcitonin, and ferritin have been found to be elevated in both COVID-19 and diabetes.^8^

Diabetic COVID-19 patients have had a poorer outcome in several hospital-based observational studies.^9^^–^^11^ Although the short-term mortality of patients with COVID-19 infection and hyperglycemia with and without preexisting diabetes has been well documented, data around longer-term outcomes is now emerging. It remains unclear whether patients have increased susceptibility to developing diabetes or related complications after recovery from the infection. To gain a better understanding around this, a registry has been established that will collect data on COVID-19–related diabetes as a part of the COVID-19IAB project.^12^^,^^13^ Clearly the diabetogenic effect of SARS CoV-2 needs to be elaborated upon and studied further.

In the present study, we followed patients with COVID-19 who had presented with hyperglycemia without prior history of diabetes to evaluate the need for oral hypoglycemic agents (OHAs) and insulin in these patients at 6 months. We also evaluated the risk factors associated with persistence of hyperglycemia.

## MATERIALS AND METHODS

The current study recruited a historical cohort of subjects hospitalized between January and June 2021 with COVID illness, and they were subsequently followed for a period of 6 months.

Patients who presented to the hospital with features of COVID-19 illness and with new-onset hyperglycemia (random blood sugar > 140 mg/dL) were included in the study after they provided informed and verbal consent for participation obtained telephonically. Those individuals who developed hyperglycemia during hospital stay after administration of glucocorticoids as part of their treatment were not included in our study. Those who were receiving long-term glucocorticoids for comorbid conditions were excluded.

The disease was classified as moderate (presence of dyspnea, hypoxia, fever or cough with an oxygen saturation between 90% and 93% on room air or respiratory rate between 24 and 30 breaths per minute) or severe (clinical features of pneumonia as well as respiratory rate more than 30 breaths per minute or severe respiratory distress or oxygen saturation < 90% on room air at the time of presentation) as per guidelines issued by Ministry of Health and Family Welfare, government of India.^13^ Participants who had previously existing diabetes, who were receiving long-term steroid therapy, or who succumbed to illness in the hospital were excluded from the study.

The participants were evaluated for the following:

1. Nature of presenting complaints and duration of symptoms
2. History of comorbidity prior to infection with COVID-19
3. Records of blood glucose during hospital stay
4. Severity of illness
5. Duration of hospital stay
6. Details regarding type of steroids
7. Details regarding therapeutic agents received during hospital stay (antibiotics and supplements, plasma therapy, etc.)
8. Details of therapeutic agents prescribed at the time of discharge with particular attention to antihyperglycemic agents

At the time of discharge, the patients were asked to monitor their blood glucose regularly at home. The participants were followed telephonically every month after discharge for 6 months from the onset of symptoms of the illness. Those who reported a self-monitored random blood glucose (SMBG) of > 140 mg/dL at the end of 6 months or were on antidiabetic therapy (oral or injectable) were included in the persistent hyperglycemia group at outcome.

The data collected as a part of this study was analyzed using Stata software (version 17; StataCorp, College Station, TX). The proportion of patients who continued to have hyperglycemia at the end of follow-up period is presented as number (percentage). The independent exposure variables at the time of admission were compared between the two groups (those who continued to have hyperglycemia at end of follow-up and those who became euglycemic without therapy) using χ^2^ test or the unpaired *t* test as appropriate. A multivariable logistic regression analysis was conducted to determine adjusted odds ratios for various risk factors. Variables found significantly associated with the outcome were included in the model. In addition, scientifically appropriate variables were forced into the model. Associations are reported as odds ratios and *P*-values < 0.05 are considered significant.

The study was initiated after ethical clearance was received from the Institutional Ethics Committee for Human Research. Each participant went through the informed consent process and was included only after explicitly documenting verbal consent telephonically. Adequate care was taken for data security and respect for individual privacy. The data sheets were not shared with anyone not directly involved with the study. The study team respected participant autonomy, and the subjects were free to leave the study at any time.

## RESULTS

**Figure 1.**
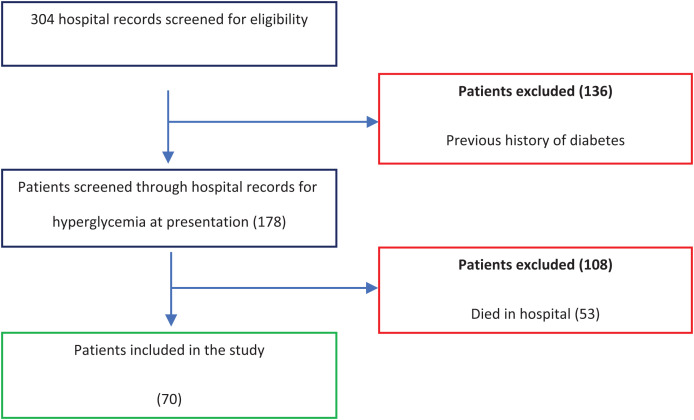
Flow diagram for the study.

A flow diagram of the study is presented in Figure 1. A total of seventy adult participants were included in the study, with a mean age of 52.2 years (±17.4). Of these, 36 (51%) were males and 34 (49%) females.

### Clinical profile.

Of seventy patients, 57 (84.3%) had fever, 40 (57.1%) had shortness of breath, 46 (65.7%) had cough, six (8.5%) had gastrointestinal discomfort, four (5.7%) had headache, and two (2.8%) had chest pain on initial presentation to the hospital. The median respiratory rate at presentation was 20 breaths/minute (interquartile range [IQR]: 18–24) and the mean oxygen saturation was 89.1% (SD ± 8.9%).

**Figure 2.**
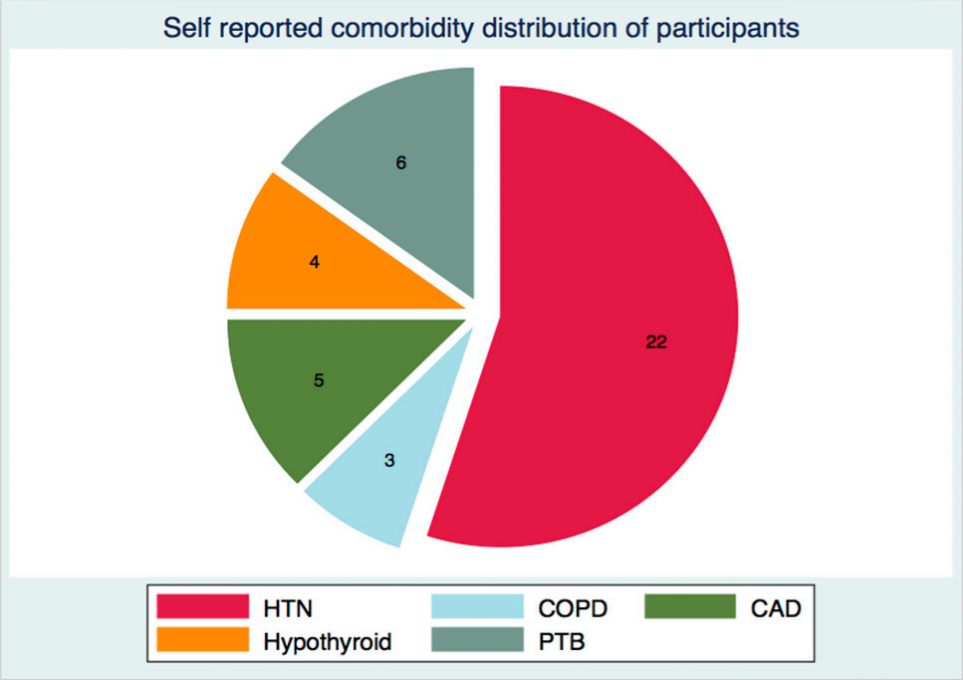
Comorbidity profile of participants. Figure depicting a pie chart of comorbidity profile of participants. HTN = hypertension, COPD = Chronic Obstructive Pulmonary Disease, CAD = Coronary Artery Disease, PTB = pulmonary tuberculosis.

Forty participants shared information regarding their comorbidity profile. The most common comorbid illness reported by the participants was hypertension. The distribution of comorbidities is presented in Figure 2.

### Laboratory profile.

The mean hemoglobin of patients on admission was 11.8 (SD ± 1.6) g/dL. The mean total leukocyte count was 8,412.8 (SD ± 3,757.6)/µL. The median blood glucose on presentation was 176 mg/dL (IQR: 158–204). The median urea on admission was 32.5 mg/dL (IQR: 24–48).

### Persistent hyperglycemia at 6 months.

**Figure 3.**
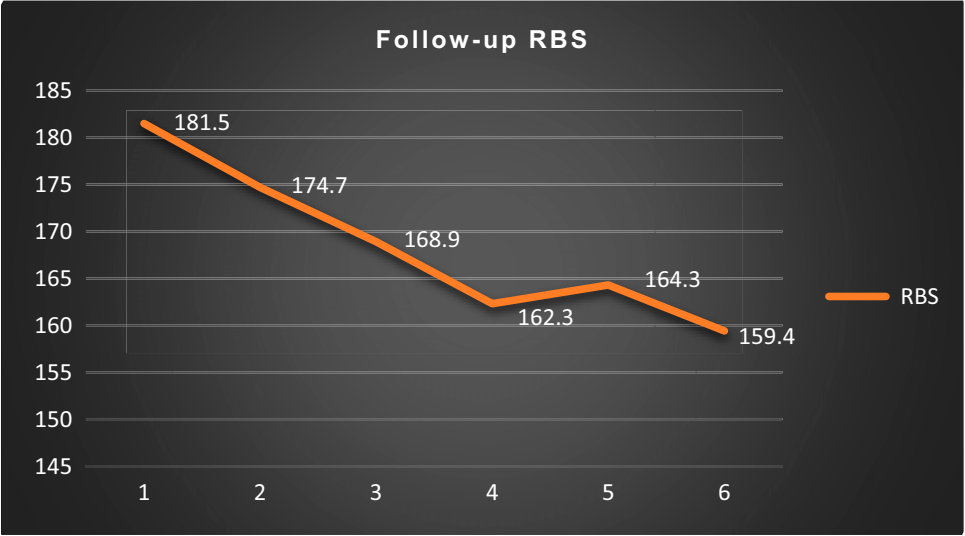
Follow-up random blood sugar (RBS) of participants. Line diagram showing the follow up RBS of patients.

Among the 70 nondiabetic participants presenting with hyperglycemia, as many as 54 (77%) reported persistent hyperglycemia (SMBG > 140 mg/dL) at the end of 6-month follow-up. Of these 54 participants, 24 (44%) were receiving oral hypoglycemic agents at 6 months, and none were receiving insulin. The self-monitored random blood glucose as reported by the patients is presented in Figure 3. A decline in mean blood glucose over the follow-up period is noted (mean blood glucose at presentation, 194; at 6 months, 160 mg/dL; paired *t* test, *P* = 0.0001).

### Factors associated with persistent hyperglycemia.

The demographic profile and presenting features are compared in patients who remained hyperglycemic with those who had reverted to euglycemia at the end of 6 months (Table 1).

**Table 1:** Comparison of profile and presenting complaints between groups

Variable	Persistent hyperglycemia at 6 months (RBS > 140), *n* = 54	Euglycemia at 6 months (RBS <140), *n* = 16	Total, *N* = 70	*P*-value
Age	54.2 (± 17.1)	45.2 (± 16.7)	52.2 (± 17.3)	0.07
Sex				
Male	29 (53.7)	7 (43.8)	36 (51.5)	0.5
Female	25 (46.3)	9 (56.2)	34 (48.5)	
Clinical presentation				
Fever (*N* = 57)	43 (79.6)	14 (87.5)	57 (81.4)	0.5
Dyspnea (*N* = 40)	30 (55)	10 (62.5)	40 (57.1)	0.6
Cough	34 (62.9)	12 (75)	46 (65.7)	0.4
Headache	3 (5.5)	1 (6.2)	4 (5.7)	0.9
GI discomfort	6 (11.1)	0 (0)	6 (8.5)	0.2
Disease severity				
Mild	13 (68.4)	6 (31.6)	19 (27.3)	
Moderate	18 (75.0)	6 (25.0)	24 (34.3)	0.3
Severe	23 (85.2	4 (14.8)	27 (38.6)	
Comorbidity				
Previous MI	1 (1.8)	1 (6.2)	2 (2.8)	0.4
Hypertension	18 (33.3)	4 (25)	22 (31.4)	0.5
Hypothyroidism	4 (7.4)	0 (0)	4 (5.7)	0.3
CAD	3 (5.6)	2(12.5)	5 (7.1)	0.3
COPD	3 (5.6)	0 (0)	3 (4.2)	0.3
PTB	3 (5.6)	3 (18.7)	6 (8.5)	0.09
Lifestyle factors				
Smoking (*N* = 3)	2 (3.7)	1 (6.2)	3 (4.2)	0.7
Alcohol use (*N* = 6)	5 (9.2)	1 (6.2)	6 (8.5)	0.7
Family history of DM	7 (12.9)	1 (6.2)	8 (11.4)	0.6
Disease severity				
Mild	13 (24.1)	6 (37.5)	19 (27.1)	
Moderate	18 (33.3)	6 (37.5)	24 (34.3)	0.4
Severe	23 (42.6)	4 (25)	27 (38.6)	
Resp. rate (breaths/min)	21.7 (± 5.7)	20.3 (± 5.2)	21.4 (± 5.6)	0.3
SpO_2_ (% saturation)	88.5 (± 9.2)	91.4 (± 7.4)	89.1 (± 8.9)	0.9
Laboratory parameters				
Hb (gm/dL)	11.7 (± 1.6)	12.0 (± 1.5)	11.8 (± 1.6)	0.6
TLC (cells/mm^3^)	8,727.7 (± 3,839.2)	7,350 (± 3,362.7)	8,412.8 (± 3,757.6)	0.2
Urea (mg/dL)	40.4 (± 20.8)	32.3 (± 19.6)	38.6 (± 20.6)	0.2
Albumin (g/dL)	3.5 (± 0.4)	3.8 (± 1.3)	3.6 (± 0.4)	0.9
Sodium (mEq/L)	137.1 (± 5.5)	138.6 (± 6.4)	137.4 (± 5.7)	0.8
Potassium (mEq/L)	4.2 (± 0.6)	4.6 (± 0.6)	4.3 (± 0.6)	0.9
Treatment				
Ivermectin (*N* = 12)	11 (20.3)	1 (6.2)	12 (17.1)	0.2
Steroid (*N* = 58)	45 (83)	13 (81.2)	58 (82.8)	0.8
No steroid	9 (16.7)	3 (18.8)	12 (17.1)	
Dexamethasone	41 (75.9)	12 (75)	53 (75.7)	
Methylprednisolone	4 (7.4)	1 (6.3)	5 (7.1)	
Cum equiv dose	435.2 (± 51.4)	199.3 (± 40.1)	381.3 (± 42.3)	0.02*
RBS (mg/dL) at presentation	207.9 (± 76.3)	152.1 (± 12.0)	195 (± 71.1)	0.005*

Alb = albumin; CAD = coronary artery disease; COPD = chronic obstructive pulmonary disease; Cum equiv dose = cumulative equivalent dose of steroid; GI = gastrointestinal; Hb = hemoglobin; HTN = hypertension; MI = myocardial infarction; PTB = pulmonary tuberculosis; RBS = random blood sugar/glucose; Resp. = respiratory; SpO2 = oxygen saturation; TLC = total leukocyte count. Categorical variables are expressed as number (percentage) and *P*-values. Continuous variables are expressed as mean (± SD), and *P*-values are based on *t* test. Values for sex are defined for females.

* *P*-values <0.05, which is a statistically significant value.

Those who had persistent hyperglycemia at 6-month follow-up also had higher blood glucose (207.9 ± 76.3 mg/dL) at presentation compared with those who had euglycemic status at 6-month follow-up (152 ± 12 mg/dL; *P* = 0.005).

Those with persistent hyperglycemia had a clinically more severe disease (higher respiratory rate, lower oxygen saturation) on simple eyeball test. However, these trends were not statistically significant.

The participants in our study were treated during the pandemic using different modalities that included steroids, antibiotics, antimalarial agents, antiviral agents, and plasma therapy. No particular therapy showed any association with persistence of hyperglycemia.

We developed multivariable logistic regression models including the parameters sequentially forwards that were found significant in the univariate analysis. Age and sex were forced into the model due to their universal confounding nature. Because steroids have been shown to be important due to their pharmacodynamics and pharmacokinetic properties, these were also included in the model. A total of 58 participants in our study received steroids (53 received dexamethasone, five received methylprednisolone). Disease severity was also included in the model. The results of the analysis are presented in Table 2.

**Table 2:** Regression analysis for persistent hyperglycemia (> 140 mg/dL)

	Univariate analysis		Multivariable analysis	
Variable	Crude OR	Significance	Adjusted OR	Significance
Risk factor				
Age	1.03 (0.99–1.06)	0.07	0.99 (0.94–1.04)	0.7
Sex	0.67 (0.21–2.06)	0.5	0.3 (0.05–1.8)	0.2
RBS	1.13 (1.06–1.21)	0.001*	1.14 (1.05–1.23)	0.001*
Cumulative steroid dose	1.002 (1.0003–1.004)	0.02*	1.001 (0.997–1.005)	0.4
Disease severity				
Mild				
Moderate	1.4 (0.36–5.28)	0.6	1.03 (0.14–7.8)	0.98
Severe	2.65 (0.63–11.16)	0.2	0.74 (0.05–10.1)	0.8

OR = odds ratio. Regression analysis of age, sex, random blood sugar/glucose (RBS), steroids, urea, and oxygen saturation (SpO_2_). RBS was significant with a *P*-value of 0.001 (< 0.05) in both univariate and multivariate analysis. Data in parentheses indicate confidence interval for the odds ratio.

* Indicates *P*-value less than 0.05 significance level.

## DISCUSSION

In our study, we recruited 70 patients of reverse-transcriptase polymerase chain reaction–proven COVID-19 who had no prior history of diabetes mellitus but were hyperglycemic at presentation. They were followed for a period of 6 months to identify persistent hyperglycemia. Of the 70 participants, 54 (77%) continued to be hyperglycemic at the end of the 6-month follow-up. Patients with persistent hyperglycemia were older and had significantly higher blood glucose at initial presentation.

### Presenting blood glucose.

Metwally et al. systematically reviewed the bidirectional nature of the relationship between COVID-19 and diabetes and discussed the possibility of COVID-19 infection inducing hyperglycemia and diabetes.^14^ They emphasized the role of continuous glucose monitoring systems during follow-up. We have explored the association that Metwally et al. proposed and followed our patients for 6 months to characterize the relationship further. A significant proportion of those with newly detected hyperglycemia continued to have persistently high blood glucose even at 6 months after onset of COVID-19 illness.

Zhang et al. retrospectively studied the outcomes in 166 adult patients (mean age 62.7 years) with or without a history of diabetes at a very early stage in the pandemic in 2020 and reported that 21 (12%) had hyperglycemia (hemoglobin A1c > 6.5%) at presentation, and those with newly detected hyperglycemia were older (68 versus 60 years).^15^ Although we did not directly compare presentations with hyperglycemia with euglycemia to enable us to make a direct comparison with the results of Zhang et al., we report similar trends at follow-up.

### Role of steroids.

The role of steroids has often evoked debate.^16^^–^^19^ Although appropriately timed steroid therapy has provided mortality benefit in severe COVID-19, its role has remained unclear in milder illness.^18^^,^^20^ Kim et al. followed 231 nondiabetic patients (median age 55 years) with lung diseases, who received steroids (median dose equivalent to 4,965 mg prednisolone; median duration 193 days).^21^ Of these, 34 (14.7%) developed steroid-induced diabetes. In our study, 58 (82.8%) patients received steroids in a median dose equivalent to 222 mg (IQR: 159–363) methylprednisolone in a median duration of 7 (IQR: 5–10) days. The persistence of hyperglycemia was not associated with the nature of steroid received, or its duration.

### Disease severity.

Fadini et al. retrospectively analyzed the data of 413 patients to evaluate the severity of COVID-19 in hyperglycemic patients.^22^ They reported poor hematological, immunological, respiratory, and radiological profile among 21 patients who were newly detected to have hyperglycemia during hospital stay. Further, higher blood glucose levels at admission were significantly linked with poorer outcomes. The authors reported that for every 36 mg/dL rise in admission blood glucose values, there was a 15% increase in severity of various parameters analyzed—more so for the newly detected hyperglycemic patients.

In an observational study, Smith et al. studied severity of COVID-19 in 184 patients (mean age 64 years, 53% males) with impaired glucose metabolism in New Jersey and found that hyperglycemia was associated with a greater need for mechanical ventilation.^23^ They reported that infection with COVID-19 was associate with new and persistent hyperglycemia during hospital stay and hypothesized that the pathogenesis involved a novel interplay with glucose metabolism.

In a multicenter analysis, Bode et al. analyzed 1,122 patients with COVID-19 in 88 American hospitals and found that 257 (22.9%) had newly diagnosed hyperglycemia.^24^ Those with hyperglycemia were older and had poorer renal profile, longer hospital stay, and greater mortality (28.8% versus 6.2%).

### Persistent hyperglycemia.

Montefusco et al. described glycemic abnormalities in 551 patients without preexisting diabetes hospitalized with COVID-19 in Italy and reported that 46% were hyperglycemic 2 months after disease onset.^3^ They concluded that COVID was associated with persistent glycometabolic abnormalities for up to 2 months. Ziyad Al-Aly et al. have characterized the postacute sequelae of COVID-19 using the national healthcare databases of the U.S. Department of Veterans Affairs among patients who survived beyond 30 days after diagnosis. They have shown a higher incidence of respiratory symptoms, neurocognitive disorders, malaise, fatigue, musculoskeletal pain and anemia. They reported an excess burden of several metabolic disorders including diabetes (hazard ratio: 8.23 [6.36–9.95]) at the end of 6-month follow up.^25^ We report persistence of similar abnormalities until 6 months.

Despite limited literature evaluating long-term glycemic status of COVID-19 patients who presented with new onset hyperglycemia, published studies concur that not only do abnormalities of glucose metabolism persist, but hyperglycemic individuals also have poorer outcome.

### Strengths and limitations.

Although several authors have reported the association between hyperglycemia and a poor short-term outcome, long-term impacts continue to be evaluated. Our study provides data on follow-up of patients with COVID-19 presenting with newly detected hyperglycemia and continuing to show persistence of elevated blood glucose after 6 months suggesting a possibility of COVID-induced diabetes. Our results allow informed decision-making around the need for continued monitoring of blood glucose. An association of disease severity with persistence of hyperglycemia is not explained by factors such as stress hyperglycemia and steroid-induced hyperglycemia that were observed during the pandemic. Our study brings into focus, the possibility of a surge in diabetes in the post COVID-19 era.

Our analysis includes a retrospectively recruited cohort for the study purpose. Although glycosylated hemoglobin would have been a worthwhile addition to the patient profile, and would have allowed us to characterize unmasking of previously existing diabetes, this remains a limitation of our study. Self-monitored blood glucose values recorded randomly by home-based glucometer readings are not as accurate as scheduled standardized laboratory evaluation. Although this remains a limitation of our study, it must be considered that the patients were followed when health systems were operating at less than ideal conditions in an atmosphere of fear and anxiety during the pandemic situation. Follow-up investigations (e.g., HbA1C, C peptide, interleukin-6), elucidation of cytokines and antibodies involved in the immune response to COVID would be helpful in evaluation of the pathogenesis of hyperglycemia.

New-onset hyperglycemia in COVID-19 is relatively less explored area of research. A prospective cohort study with multicentric data can give us a better perspective for broader generalization and extrapolation. A longer follow-up of normoglycemic patients to determine whether they are more prone to developing diabetes in the future may shed additional light on the evolving pathogenesis of the COVID-19 disease and rapidly disappearing divide between communicable and noncommunicable disease epidemiology.

## CONCLUSION

In our retrospective cohort study, we found that three of four nondiabetic patients with COVID-19 infection who presented with hyperglycemia reported persistent glycometabolic abnormalities 6 months after disease onset. COVID-19 infection has had a huge impact on humanity, and many long-COVID-19 effects are being uncovered. COVID-19-induced diabetes is one such complication that may pose serious adverse effects on public health.
